# Enhancing diagnostics: ChatGPT-4 performance in ulcerative colitis endoscopic assessment

**DOI:** 10.1055/a-2542-0943

**Published:** 2025-03-14

**Authors:** Asaf Levartovsky, Ahmad Albshesh, Ana Grinman, Eyal Shachar, Adi Lahat, Rami Eliakim, Uri Kopylov

**Affiliations:** 1 26744 Gastroenterology, affiliated with Tel Aviv University, Sheba Medical Center, Tel Hashomer, Israel

**Keywords:** Endoscopy Lower GI Tract, Inflammatory bowel disease, Diagnosis and imaging (inc chromoendoscopy, NBI, iSCAN, FICE, CLE...), Quality and logistical aspects, Image and data processing, documentatiton

## Abstract

**Background and study aims:**

The Mayo Endoscopic Subscore (MES) is widely utilized for assessing mucosal activity in ulcerative colitis (UC). Artificial intelligence has emerged as a promising tool for enhancing diagnostic precision and addressing interobserver variability. This study evaluated the diagnostic accuracy of ChatGPT-4, a multimodal large language model, in identifying and grading endoscopic images of UC patients using the MES.

**Patients and methods:**

Real-world endoscopic images of UC patients were reviewed by an expert consensus board. Each image was graded based on the MES. Only images that were uniformly graded were subsequently provided to three inflammatory bowel disease (IBD) specialists and ChatGPT-4. Severity gradings of the IBD specialists and ChatGPT-4 were compared with assessments made by the expert consensus board.

**Results:**

Thirty of 50 images were graded with complete agreement among the experts. Compared with the consensus board, ChatGPT-4 gradings had a mean accuracy rate of 78.9% whereas the mean accuracy rate for the IBD specialists was 81.1%. Between the two groups, there was no statistically significant difference in mean accuracy rates ( *P* = 0.71) and a high degree of reliability was found.

**Conclusions:**

ChatGPT-4 has the potential to assess mucosal inflammation severity from endoscopic images of UC patients, without prior configuration or fine-tuning. Performance rates were comparable to those of IBD specialists.

## Introduction

Endoscopic evaluation is the key tool for assessing and managing inflammation in ulcerative colitis (UC) patients. This assessment has a key role in clinical practice because endoscopic remission is a therapeutic objective associated with long-term clinical remission and reduced colectomy risk 1 2 . The Mayo Endoscopic Subscore (MES) is a widely utilized scoring measure for endoscopic disease activity in UC 3 . The MES assigns a 4-point endoscopic severity score based on the most severely inflamed areas with 0, 1, 2, or 3 points given for normal, mild, moderate, or severe disease, respectively. However, scoring systems such as the MES, which are entirely based on subjective interpretation of images, face the challenge of interobserver variability 4 5 . This issue can lead to discrepancies in severity assessments, especially among non-specialist gastroenterologists. Variability underscores the need for accurate and reproducible diagnostic tools to aid in clinical decision-making.

Artificial intelligence (AI) has recently emerged as a promising tool for enhancing diagnostic precision and addressing interobserver variability among endoscopists. These tools can incorporate machine learning and deep learning methods for image analysis, enabling more accurate evaluation of disease severity 6 7 8 . Most recently, large language models (LLMs) such as ChatGPT (OpenAI) have shown considerable progress and been widely explored in the medical landscape, including gastroenterology. Recent publications have demonstrated ChatGPT application in answering common questions about colonoscopy and generating accurate guideline-based recommendations for inflammatory bowel disease (IBD) based on the European Crohn’s and Colitis Organization guidelines 9 10 11 . In addition, ChatGPT effectiveness and potential to assist in clinical severity assessment of acute UC presentations in the emergency room was recently showcased 12 . The latest version of this LLM is ChatGPT-4, a multimodal model able to accept input data in the form of text or images.

This study aimed to evaluate the accuracy and reproducibility of ChatGPT-4 in identifying and grading endoscopic images of UC patients using the MES as a reference standard, without prior configuration or fine-tuning.

## Patients and methods

This pilot study included the task of grading severity (0–3) of endoscopic images based on the MES. Real-world endoscopic images were obtained for severity assessment and reviewed by an expert consensus board (A.La., R.E., U.K.). Only images that were uniformly graded by the consensus board were subsequently provided to three IBD specialists (A.A., A.G., E.S.) and ChatGPT-4 in three separate sessions. MES gradings of ChatGPT-4 and the IBD specialists were compared with assessments made by the expert consensus board. Each image was submitted to ChatGPT three times, each to a new session.

The exact prompt used for each session of ChatGPT-4 was as follows: “I need you to be an expert gastroenterologist in the field of IBD and endoscopy. You will assist in analyzing colonoscopy images of UC patients and grading them based on the Mayo Endoscopic Score. I will provide a total of 30 images and you will return a specific score for each image (0/1/2/3)”.

Statistical analysis included two steps. First, mean proportions of accurate MES assessments made by ChatGPT-4 and the IBD specialists (compared with the expert consensus board) were calculated. Second, we performed a statistical *t* test analysis to compare mean accurate response rates and intra-class correlation of agreement analysis to evaluate the degree of reliability between these two groups.

## Results

**Fig. 1:**
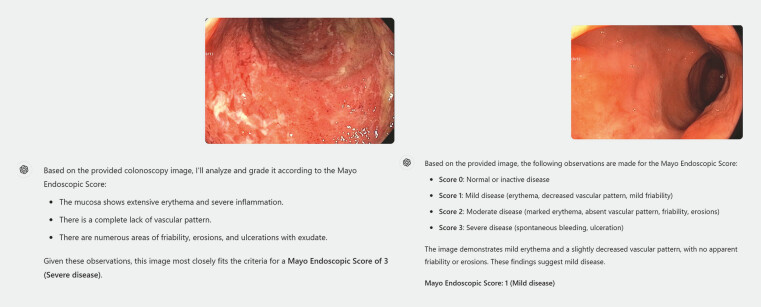
ChatGPT-4 responses of MES assessments.

Fifty endoscopic images were initially evaluated by the expert consensus board. Of those, 30 images (60%) were graded with complete agreement of MES severity among the expert consensus board. These included 12, 7, 3, and 8 images with severe, moderate, mild, and inactive disease, respectively. ChatGPT-4 provided answers for each image in all sessions. For each image, ChatGPT-4 elaborated and depicted the relevant mucosal findings and concluded a final MES assessment based on those findings ( Fig. 1 ).

**Fig. 2:**
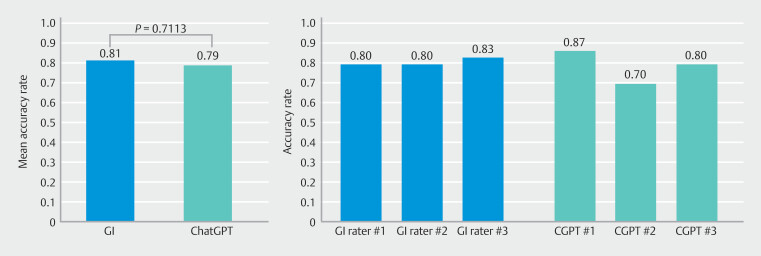
Mean accuracy rates between the IBD specialists and ChatGPT-4.

Compared with the consensus board, ChatGPT-4s gradings were accurate in 26 of 30 (86.7%), 21 of 30 (70%) and 24 of 30 cases (80%), with a mean accuracy rate of 78.9%. IBD specialists were accurate in 24 of 30 (80%), 24 of 30 (80%), and 25 of 30 cases (83.3%), with a mean accuracy rate of 81.1% ( Fig. 2 ). There was no statistically significant difference in mean accuracy rates between the two groups ( *P* = 0.71). A high degree of reliability was found between the IBD specialists and ChatGPT-4. The average measure intra-class correlation coefficient of absolute agreement between the groups was 0.918 (95% confidence interval 0.876–0.946, F = 12.1, *P* <0.001).

## Discussion

This unique study challenges the dynamic role of AI in endoscopy and hints at a potential for ChatGPT-4 as a tool for enhancing diagnostic assessment. ChatGPT-4 was effective and accurate in grading endoscopic images of UC patients using a well-established endoscopic severity score. The comparable performance of ChatGPT-4 to trained gastroenterologists, with mean accuracy scores of 0.79 and 0.81, respectively, suggests that LLM models can effectively assist in crucial image-based clinical decision-making processes.

Application of AI in endoscopy has been reported to provide accurate outputs, exemplified by the likes of computer-aided diagnosis for polyp detection and AI-assisted colonoscopy for UC monitoring 13 14 . Nonetheless, AI-based models developed for these purposes utilize extremely large data sets of images and videos for training and validating diagnostic capabilities to approximate human performance. One of the significant advantages of using LLMs like ChatGPT-4 is their ability to provide consistent and unbiased evaluations, which can help mitigate variability in human-based grading. For the reasons discussed above, an LLM-based solution is particularly valuable in this clinical field.

To the best of our knowledge, this is the first study to input real-world endoscopy images in LLMs for the purpose of clinical assessment. Use of ChatGPT-4 is an accessible, reproducible, and time-sparing method for analyzing images of clinical importance. A notable advantage was use of a concise prompt, without prior configuration or fine-tuning. The significance of this virtue is ease of access to an accurate, untrained LLM, compared with existing deep learning methods specifically fine-tuned and trained for endoscopic assessments.

Despite the promising findings, the study is not without limitations. The small sample size consisting of single endoscopic frames requires further validation across larger cohorts to increase generalizability of our findings and strengthen external validity. It is vital to emphasize that it is not possible to appreciate the broad severity of mucosal inflammation based on a single endoscopic image. This was planned as a proof-of-concept study and by no means implies that a single image can replace a thorough endoscopic examination. Moreover, future implementation of a video-based analysis would allow continuous assessment of the mucosal surface and potentially enhance diagnostic accuracy.

Utilization of the MES is simple and straightforward, yet considerable drawbacks include lack of extent of lesions and referring to the worst visible lesion (regardless of lesions in other segments). Perhaps more comprehensive scoring systems such as the UC endoscopic index score or the modified MES could address additional issues. Another important limitation is the inconsistent responses of ChatGPT-4 when presented with the same prompts and endoscopic images. This flaw is due to inherent randomness of the model and underscores the need for validating its outputs, as with any AI-generated output. Future AI-based development may include stricter control over generation parameters or specific settings to minimize this randomness.

## Conclusions

In conclusion, ChatGPT-4 has the potential to objectively evaluate disease activity and maintain reproducibility, thus enhancing the overall quality of our endoscopic assessments. Although this concept warrants further research and validation, its intriguing abilities can revolutionize the way we evaluate and monitor our patients.
